# Frequency-adjusted daratumumab-based regimen versus bortezomib/dexamethasone in newly diagnosed AL amyloidosis: a matched-cohort study

**DOI:** 10.1080/07853890.2026.2617767

**Published:** 2026-01-21

**Authors:** Wanting Zheng, Meilan Zhou, Yan Xing, Jin Zhao, Baojian Liu, Wenjing Li, Shiren Sun

**Affiliations:** Department of Nephrology, Xijing Hospital, The Fourth Military Medical University, Xi’an, China

**Keywords:** AL amyloidosis, daratumumab, immunotherapy

## Abstract

**Background:**

The optimal dosing schedule for daratumumab (Dara) in newly diagnosed systemic light-chain (AL) amyloidosis requires refinement. This real-world study compared the efficacy and safety of a frequency-adjusted, cyclophosphamide-free Dara-based regimen with bortezomib/dexamethasone (BD).

**Methods:**

We included newly diagnosed AL amyloidosis patients treated between January 2018 and December 2024, with follow-up data censored in August 2025. Using 1:1 propensity score matching based on age, cardiac stage, dFLC, and organ function, we compared hematologic/organ responses and survival. Survival was analyzed with Kaplan-Meier and log-rank tests. The Dara-based group received a cyclophosphamide-free regimen with an adjusted schedule (biweekly in cycle 1, then monthly) and a response-guided treatment duration.

**Results:**

A total of 105 patients were included. After propensity score matching, 60 patients (30 per group) were selected for comparative analysis. The Dara-based group achieved significantly higher hematologic complete response (CR) rates at 6 months (57% vs. 27%, *p* = 0.018) and 12 months (63% vs. 27%, *p* = 0.002), with a markedly shorter median time to CR (61 vs. 120 days, *p* = 0.010). Organ responses were also superior: cardiac response 52% vs. 24% (*p* = 0.037) and renal response 56% vs. 27% (*p* = 0.037). No significant survival difference was observed during follow-up, and the safety profile was comparable between groups.

**Conclusion:**

A frequency-adjusted Dara-based regimen induces significantly faster and deeper hematologic and organ responses compared to BD in newly diagnosed AL amyloidosis which offers a promising personalized approach to reduce treatment burden and adverse events while maintaining high efficacy, supporting its integration into clinical practice for a broader patient population.

## 1. Introduction

Light chain (AL) amyloidosis is a life-threatening disorder caused by organ deposition of misfolded immunoglobulin light chains, leading to progressive organ dysfunction [1]. Treatment aims to eliminate the underlying clonal plasma cells, largely following strategies for multiple myeloma. While therapeutic advances over the past four decades have improved clinical outcomes [2,3], the prognosis remains dismal for patients with advanced cardiac involvement, exemplified by a two-year survival rate of less than 30% in Mayo stage III disease [4]. The introduction of the anti-CD38 monoclonal antibody daratumumab (Dara) represents a landmark advancement [5]. The phase III ANDROMEDA trial established that Dara combined with cyclophosphamide, bortezomib, and dexamethasone (D-VCd) significantly outperformed VCd alone, demonstrating superior hematologic complete response (CR) (59.5% vs. 19.2%) and organ response rates (cardiac: 42%; renal: 53%) [6]. Consistent with this, multiple studies confirm that Dara-based regimens induce rapid, deep hematologic responses and organ improvement [5,7,8], solidifying their role as a cornerstone of frontline therapy for newly diagnosed AL amyloidosis [9].

Nevertheless, key questions persist regarding the practical implementation of Dara. The extended treatment schedules adopted from multiple myeloma may not be universally necessary for AL amyloidosis due to its characteristically low tumor burden, and their application could contribute to increased treatment burden and toxicity without clear additional benefit. Furthermore, the generalizability of findings from pivotal trials such as ANDROMEDA is constrained by the exclusion of high-risk patients, including those with Mayo stage IIIb disease, creating an evidence gap relevant to real-world clinical practice. Notably, evidence suggests that cyclophosphamide-free regimens based on bortezomib and dexamethasone (BD) may yield comparable or even superior hematologic and cardiac outcomes [10]. Recently, a prospective phase II study demonstrated promising efficacy and safety of Dara in combination with BD (Dara-BD) even in advanced-stage patients, supporting the feasibility of cyclophosphamide-free regimen [7].

Despite these developments, high-quality real-world comparisons between Dara-based therapies and conventional BD remain scarce. There is a growing need to explore optimized Dara-based strategies that minimize overtreatment and economic burden while preserving efficacy. Therefore, we conducted this real-world study to evaluate the efficacy and safety of a novel, frequency-adjusted, cyclophosphamide-free Dara-based regimen compared to conventional BD in patients with newly diagnosed AL amyloidosis, using propensity score matching for a robust comparison.

## 2. Patients and methods

### 2.1. Patients and treatment regimens

**Figure 1.**
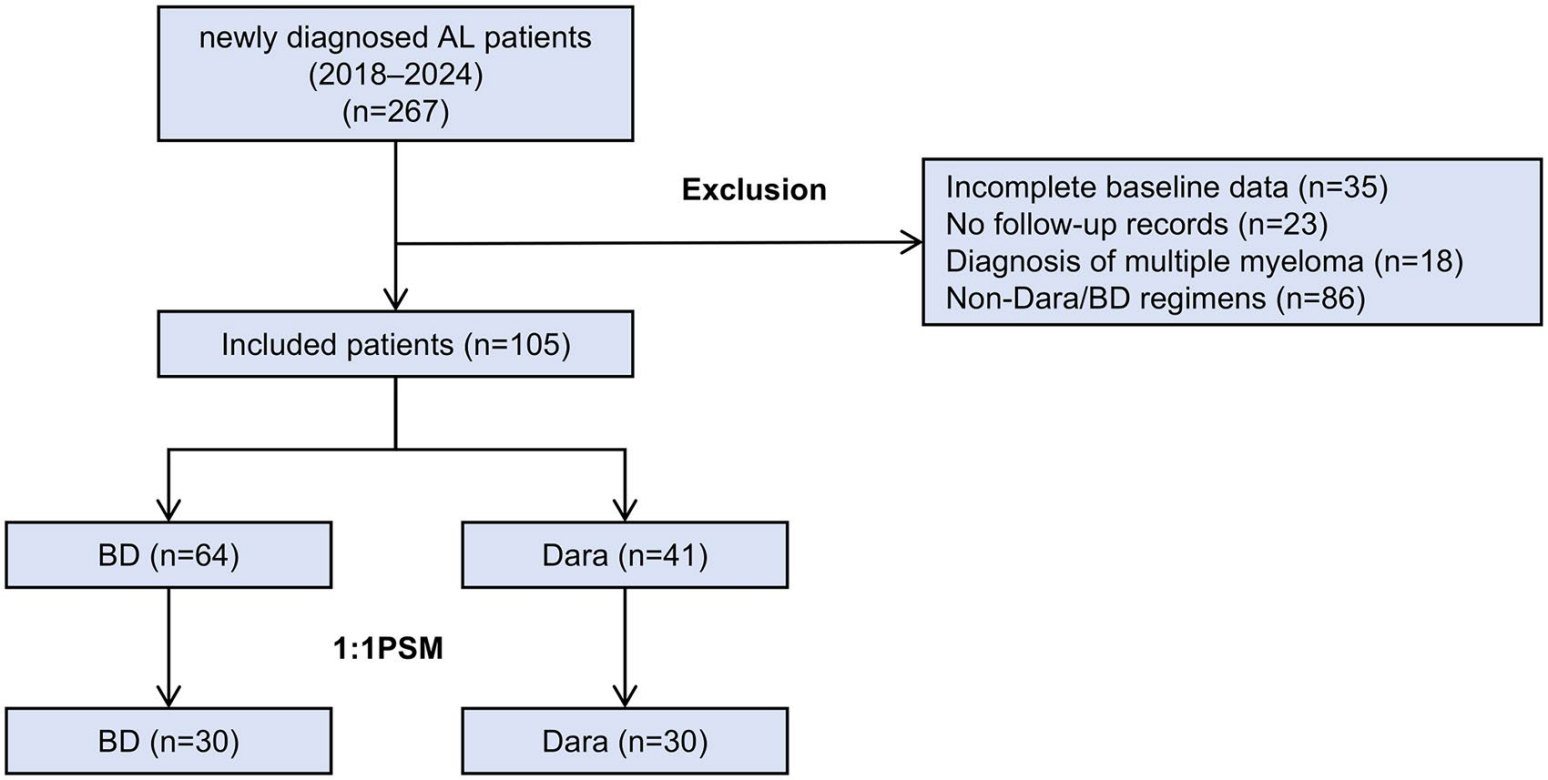
Study cohort selection and matching. Abbreviations: AL: systemic light chain amyloidosis; BD: bortezomib and dexamethasone; Dara: daratumumab-based regimen; PSM: propensity score matching.

This retrospective cohort study consecutively enrolled newly diagnosed, histologically confirmed AL amyloidosis patients with at least one organ involvement at Xijing Hospital between January 2018 and December 2024. The data lock point for follow-up was August 2025, and the data analysis for this study was conducted from September to October 2025. The diagnosis of systemic AL amyloidosis required all of the following criteria: (1) tissue biopsy with Congo red staining demonstrating amyloid deposits; (2) confirmation of AL type by immunofluorescence and/or laser microdissection with mass spectrometry; (3) evidence of a monoclonal plasma cell proliferative disorder. Exclusion criteria included: (1) localized, hereditary, or secondary amyloidosis; (2) meeting the International Myeloma Working Group (IMWG) diagnostic criteria for multiple myeloma; (3) lack of complete pathological documentation; (4) insufficient baseline clinical or laboratory data, including serum free light chains, immunofixation electrophoresis, and immunoglobulin profiles; and (5) a follow-up duration of less than six months, unless the patient experienced all-cause mortality within this period. The patient selection process is summarized in Figure 1. Patients in the BD group received subcutaneous bortezomib (1.3 mg/m^2^ on days 1, 8, 15, and 22 of each 28-day cycle) and oral dexamethasone (40 mg weekly, reduced to 20 mg in cases of poorly controlled diabetes). Those in the Dara-based group received intravenous Dara (16 mg/kg) every two weeks during the first month and every four weeks thereafter. Treatment was intended to be discontinued once the patient achieved a hematologic very good partial response (VGPR) or better together with a relevant organ response, based on physician assessment and patient agreement, and could be continued for up to 24 months in the absence of disease progression. At the physician’s discretion, bortezomib and dexamethasone could be added to Dara following the same schedule as the BD group.

### 2.2. Definition

The primary endpoint was the rate of hematologic complete response as defined by the International Society of Amyloidosis (ISA) consensus criteria [11] and updated recommendations [12] (see Supplemental Table S1 for detailed criteria). For patients who died prior to the first assessment, response was determined from the last available evaluation. Secondary endpoints included hematologic overall response rate, cardiac and renal organ response rates (Supplemental Table S2), major organ deterioration–progression-free survival (MOD-PFS) [13], defined as time from initiation to hematologic progression, major organ deterioration [cardiac or renal], or death from any cause), overall survival, time to CR, and safety (assessed by CTCAE v5.0).

### 2.3. Response assessment

For hematologic response evaluation, the evaluable population was defined as patients who received at least one dose of the study therapy and had at least one post-baseline serum free light chain assessment. Hematologic and organ responses were assessed at two predefined landmark time points: 6 months and 12 months after treatment initiation. To ensure data quality and interpretability, patients who were alive but lost to follow-up before the start of the 6-month assessment window were excluded from the study during the initial screening phase, as stipulated in the exclusion criteria. For the enrolled patients, a permissible time window of ±4 weeks was applied to account for real-world variations in follow-up schedules. Assessments conducted within this window were considered valid for the respective landmark analysis. For patients with multiple evaluations within the window, the assessment closest to the exact landmark date was used. For patients who died before reaching the beginning of a landmark time window, the last available on-treatment response assessment prior to death was used for that time point analysis. This approach was adopted to include informative efficacy data from these high-risk patients and to avoid bias from informative censoring.

### 2.4. Statistical analysis

To minimize confounding, 1:1 propensity score matching was performed using nearest-neighbor method (caliper width = 0.03). Matching covariates included age, sex, light-chain type, and key organ parameters: alkaline phosphatase, 24-hour proteinuria, eGFR, dFLC, NT-proBNP, cTnI, LVEF, and IVS thickness. Analyses used SPSS 25.0. Continuous data are presented as mean ± SD or median [IQR] and compared with t-tests or Mann-Whitney U tests. Categorical data are presented as n (%) and compared with *χ*^2^ or Fisher’s exact tests. MOD-PFS was analyzed by Kaplan-Meier method with log-rank test. A two-tailed *p* < 0.05 was considered significant.

### 2.5. Ethical approval

This study was reviewed and approved by the Ethics Committee of Xijing Hospital (Approval No. XJLL-KY-20252225). The Ethics Committee of Xijing Hospital waived the requirement for informed consent due to the retrospective nature of this study, which involved minimal risk to participants. All procedures were performed in accordance with the ethical standards of the institutional research committee and with the 1964 Helsinki Declaration and its later amendments.

## 3. Results

### 3.1. Baseline characteristics

A total of 105 newly diagnosed AL amyloidosis patients were included. The median age was 58 years (IQR, 54–65), and 67.6% were male. Most patients had *λ* light chain involvement (85.7%), with high rates of cardiac (66.7%) and renal (91.4%) involvement; 58.1% had multi-organ involvement. Of the entire cohort, 64 patients (61.0%) received BD and 41 (39.0%) received a DARA-based regimen as initial treatment. Within the Dara-based group, 28 (68.3%) received Dara-BD combination and 13 (31.7%) received Dara monotherapy. The baseline characteristics of all patients are summarized in Table 1. After 1:1 propensity score matching, 60 patients (30 per group) were included in the comparative analysis. As shown in Table 2, no significant differences in baseline characteristics were observed between the matched groups.

**Table 1.** Baseline characteristics of the entire study cohort (*N* = 105).

	All patients (*n* = 105)	Dara-based (*N* = 41)	BD (*N* = 64)	*p*
Male, *n* (%)	71 (68)	27 (66)	44 (69)	0.924
Age, years, median (IQR)	59 (54, 66)	59 (54, 68)	59 (54, 66)	0.745
Involved light-chain type				1
*λ*	90 (86)	6 (15)	9 (14)	
*κ*	15 (14)	35 (85)	55 (86)	
ALP, UI/L	68 (50, 93)	79 (62, 105)	59 (34, 89)	0.002
Proteinuria, mg/24 h	2739 (1358, 5740)	2509 (1571, 3983)	3134 (1265, 5825)	0.955
eGFR, mL/min/1.73 m^2^	80 (61, 94)	80 (66, 98)	80 (58, 92)	0.321
dFLC, mg/L	100 (60, 159)	106 (54, 147)	99 (65, 166)	0.927
NT-proBNP, ng/L	767 (212, 3071)	546 (181, 2560)	880 (234, 3378)	0.331
cTnI, ng/L	30 (10, 80)	20 (10, 80)	40 (10, 70)	0.597
LVEF, %	59 (56, 61)	60 (58, 63)	58 (55, 60)	0.027
IVS thickness, mm	12 (10, 14)	12 (10, 12)	12 (10, 15)	0.848
Mayo 2004 stage				0.997
Stage I	32 (30)	13 (32)	19 (30)	
Stage II	34 (32)	13 (32)	21 (33)	
Stage IIIa	31 (30)	12 (29)	19 (29)	
Stage IIIb	8 (8)	3 (7)	5 (8)	
Mayo 2012 stage				0.712
Stage I	49 (47)	21 (51)	28 (44)	
Stage II	24 (23)	7 (17)	17 (27)	
Stage III	23 (22)	10 (25)	13 (20)	
Stage IV	9 (8)	3 (7)	6 (9)	
Organ involvement				
Kidney	96 (91)	38 (93)	58 (91)	1
Cardiac	70 (67)	27 (66)	43 (67)	0.888
Liver	6 (6)	3 (7)	3 (5)	0.676

**Table 2.** Comparison of baseline characteristics between treatment groups after propensity score matching.

	Dara-based (*N* = 30)	BD (*N* = 30)	*p*
Male, *n* (%)	20 (67)	19 (63)	1
Age, years, median (IQR)	57 (54,62)	62 (54,67)	0.293
Involved light-chain type			1
*κ*	5 (17)	6 (20)	
*λ*	25 (83)	24 (80)	
ALP, UI/L	78 (64,101)	62 (41,93)	0.057
Proteinuria, mg/24 h	2500 (1546,4245)	2206 (1080,5344)	0.626
eGFR, mL/min/1.73 m2	97 (84,104)	95 (60,103)	0.663
dFLC, mg/L	113 (74,231)	92 (51,153)	0.139
NT-proBNP, ng/L	671 (385,2943)	1588 (316,4067)	0.478
NT-proBNP, mean ± SD	2367 ± 3832	3618 ± 6228	0.353
cTnI, ng/L	30 (10,70)	40 (10,60)	0.455
LVEF, %	59 (56,60)	58 (56,60)	0.789
IVS thickness, mm	12 (11,12)	13 (9,16)	0.852
Mayo 2004 stage			0.868
Stage I	6 (20)	7 (24)	
Stage II	13 (43)	10 (33)	
Stage IIIa	9 (30)	10 (33)	
Stage IIIb	2 (7)	3 (10)	
Mayo 2012 stage			0.747
Stage I	15 (50)	13 (43)	
Stage II	6 (20)	8 (27)	
Stage III	6 (20)	8 (27)	
Stage IV	3 (10)	1 (3)	
Organ involvement			
Kidney	27 (90)	26 (87)	1
Cardiac	23 (77)	21 (70)	0.559
Liver	2 (7)	2 (7)	1

### 3.2. Treatment exposure and discontinuation

In accordance with the response-adapted strategy, the intended treatment goal was to discontinue therapy upon achieving a hematologic VGPR (or better) along with a concomitant major organ (cardiac or renal) response. As detailed in Table 3, more than half of the cohort (23/41, 56.1%) followed this pathway, receiving a median of 10 Dara administrations. Reflecting real-world practice, variations were observed: a small proportion completed the maximum 24-month course (4/41, 9.8%), while others discontinued early due to factors such as financial burden or intolerance (5/41, 12.2%). Six patients (14.6%) remained on treatment at data cutoff, and three patients (7.3%) died during follow-up after receiving a median of 2 administrations. For the entire Dara-based cohort, the median number of Dara administrations was 10 (range, 2–30).

**Table 3.** Treatment duration and discontinuation patterns in the Dara-based cohort (*N* = 41).

Discontinuation category	Patients, n (%)	Median Dara administrations (range)
Achieved response goal	23 (56.1)	10 (6–16)
Completed the 24-month treatment course	4 (9.8)	22.5 (17–30)
Early discontinuation (toxicity/financial)	5 (12.2)	5 (2–7)
Ongoing treatment at data cutoff	6 (14.6)	11.5 (9–14)
Discontinued due to death	3 (7.3)	2 (2–4)
Total Cohort	41	10 (2–30)

Within the matched cohort (*n* = 60), patients receiving the Dara-based regimen (*n* = 30) were administered a median of 10 Dara doses (range, 230). In this group, 21 patients (70%) received Dara in combination with bortezomib and dexamethasone (median bortezomib cycles: 5; range, 1–12), while 9 patients (30%) received Dara monotherapy. The matched patients treated with BD (*n* = 30) received a median of 6 cycles (range, 2–12), corresponding to a median of 24 doses of bortezomib (range, 8–48).

### 3.3. Hematologic and organ responses

Among the 41 patients who received the Dara-based regimen, the best observed hematologic responses were as follows: a CR in 68% (28/41), a VGPR in 24% (10/41), resulting in an overall response rate (ORR) of 97%.

Following propensity score matching, the comparative analysis between the 30 Dara-based and 30 BD patients demonstrated significantly deeper and more rapid hematologic responses with the Dara-based regimen. At the 6-month assessment, the rate of CR was more than twofold higher in the Dara-based group (57% [17/30] vs. 27% [8/30]; *p* = 0.018, OR 3.60, 95% CI 1.27–10.16). The ORR was also numerically superior in the Dara-based group (90% vs. 83%). The median time to CR was significantly shorter with the Dara-based regimen (61 days; IQR, 29–115) than with BD (120 days; IQR, 94–233) (*p* = 0.010). Organ response rates at 6 months were consistently higher in the Dara-based group, with a cardiac response observed in 52% (12/23) of evaluable patients versus 24% (5/21) in the BD group (*p* = 0.037, OR 3.49, 95% CI 0.96–12.73), and a renal response in 56% (15/27) versus 27% (7/26) (*p* = 0.037, OR 3.39, 95% CI 1.06–10.88) (Table 4). By the 12-month follow-up, the hematologic CR rate in the Dara-based group further increased to 63% (19/30), while the rate in the BD group remained at 27% (8/30) (*p* = 0.002, OR 4.75, 95% CI 1.62–13.94). The renal response rate also showed a significant improvement in the Dara-based group (70% [19/27]) compared to the BD group (46% [12/26]) (*p* = 0.046, OR 2.77, 95% CI 0.91–8.43). Although the cardiac response rate was numerically higher in the Dara-based group (61% vs. 43%), this difference was not statistically significant (*p* = 0.257, OR 2.07, 95% CI 0.62–6.94) (Table 5).

**Table 4.** Hematologic and organ responses at 6 months by treatment group.

	Dara-based (*N* = 30)	BD (*N* = 30)	*p*
Hematologic response, *n* (%)			
Complete response	17 (57)	8 (27)	0.018
Very good partial response	9 (30)	14 (46)	
Partial response	1 (3)	3 (10)	
No response	3 (10)	5 (17)	
Time for CR achieved (days, median (IQR))	61 (29–115)	120 (94–233)	0.010
Cardiac response, *n* (%)			
Response	12 (52)	5 (24)	0.037
No response	6 (26)	10 (48)	
Progression	5 (22)	6 (28)	
Kidney response, *n* (%)			
Response	15 (56)	7 (27)	0.037
No response	11 (40)	15 (58)	
Progression	1 (4)	4 (15)	

**Table 5.** Hematologic and organ responses at 12 months by treatment group.

	Dara-based (*N* = 30)	BD (*N* = 30)	*p*
Hematologic response, *n* (%)			
Complete response	19 (63)	8 (27)	0.002
Very good partial response	8 (27)	13 (43)	
Partial response	1 (3)	2 (7)	
No response	2 (7)	7 (23)	
Cardiac response, *n* (%)			
Response	14 (61)	9 (43)	0.257
No response	2 (9)	8 (38)	
Progression	7 (30)	4 (19)	
Kidney response, *n* (%)			
Response	19 (70)	12 (46)	0.046
No response	6 (22)	7 (27)	
Progression	2 (8)	7 (27)	

### 3.4. Survival outcome

In the overall cohort of patients receiving the Dara-based regimen (*n* = 41) with a median follow-up of 14 months (range, 3–36), the 1-year overall survival (OS) rate was 92.7% and the 1-year MOD-PFS rate was 90.2%.

**Figure 2.**
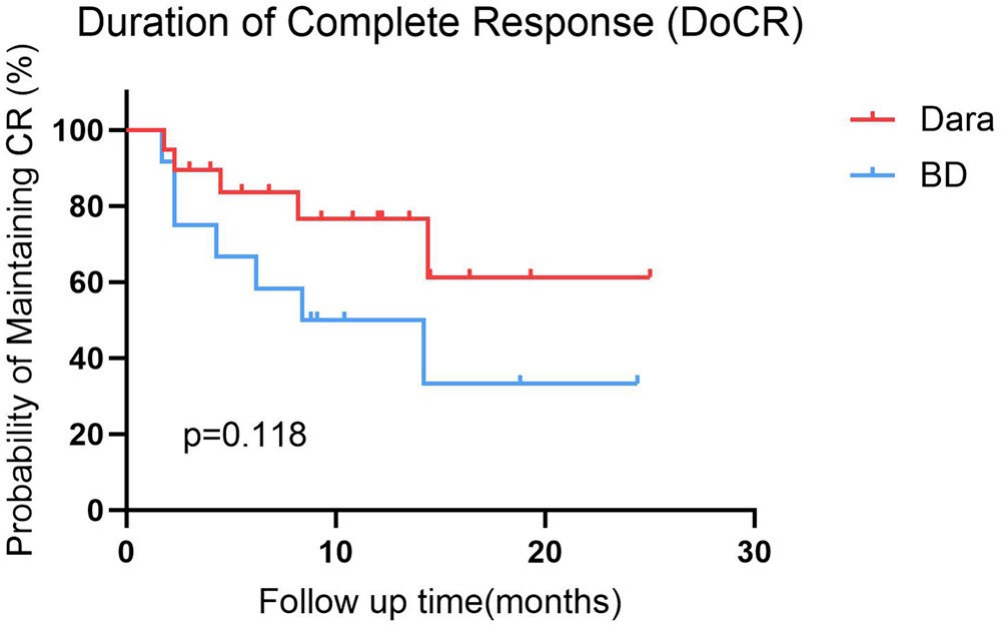
Duration of complete response in patients achieving CR.

**Figure 3.**
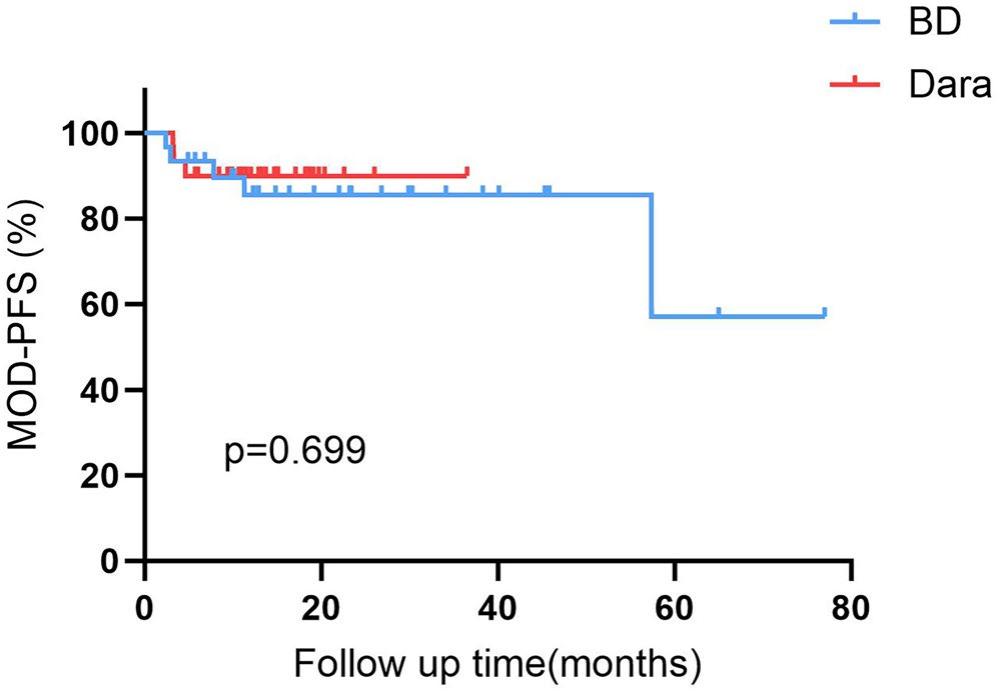
Major organ deterioration–progression-free survival (MOD-PFS) between patients receiving Dara-based regimens and BD regimens.

Within the matched cohort, the median follow-up was 14 months (range, 3–36) for the Dara-based group and 23 months (range, 3–78) for the BD group, reflecting the later introduction of Dara into clinical practice. Despite the shorter follow-up in the Dara group, a trend toward a longer duration of complete response was observed. The median CR duration was 10.8 months (IQR, 5.5–14.5) in the Dara-based group compared to 8.6 months (IQR, 4.3–14.2) in the BD group, suggesting a potential benefit in response sustainability (HR for progression or death, 0.42; 95% CI: 0.13–1.34; *p* = 0.118) (Figure 2). This favorable trend, however, did not translate into a statistically significant difference in MOD-PFS between the two groups (*p* = 0.699) (Figure 3). The 1-year MOD-PFS rates were 90% and 83.3% for the Dara-based and BD groups, respectively. OS at 1 year was 90% in the Dara-based group and 93.3% in the BD group. Five patients died during follow-up (three in the Dara-based group and two in the BD group) due to cardiac complications (*n* = 3) or multiorgan failure (*n* = 2). Notably, all deceased patients had Mayo stage III or higher disease at baseline.

### 3.5. Efficacy in patients with advanced cardiac involvement

While the efficacy of daratumumab is well-established in clinical trials, these often exclude the highest-risk patients. We specifically evaluated the efficacy of our regimen in patients with advanced cardiac involvement, defined as Mayo 2004 stage IIIa or IIIb. Within the Dara cohort, 15 patients (36.6%) belonged to this high-risk subgroup. In these patients, the regimen demonstrated considerable efficacy: the best hematologic response was CR in 60% and VGPR in 33.3%. Corresponding cardiac and renal response rates were 53.3% and 61.5%, respectively. During the follow-up period, three deaths occurred in this advanced-stage subgroup.

An exploratory analysis of three Mayo 2004 stage IIIb patients revealed deep hematologic responses (one CR, two VGPR). Notably, two of these three critically ill patients achieved a cardiac response. These individual outcomes, while not generalizable, suggest that our Dara-based regimen has the potential to benefit even the most severely affected patients for whom prognosis is historically dire.

### 3.6. Safety and tolerability

Treatment was well tolerated in both groups, with a comparable incidence and spectrum of adverse events (Table 6). The most common all-grade adverse events were edema (Dara-based 30% vs. BD 37%), diarrhea (27% vs. 30%), and fatigue (23% vs. 27%). The incidence of peripheral neuropathy was 13% in the Dara-based group and 23% in the BD group. No statistically significant differences were found for any adverse event (all *p* > 0.05). A similar total number of events was recorded (47 vs. 51). Importantly, all reported adverse events were of grade 1 or 2 severity, resolved with appropriate management, and no grade 3 or 4 events occurred.

**Table 6.** Adverse events between treatment groups.

Events	Dara-based (*N* = 30)	BD (*N* = 30)	*p*
Edema (*n*,%)	9 (30)	11 (37)	0.61
Diarrhea (*n*,%)	8 (27)	9 (30)	0.79
Constipation (*n*,%)	6 (20)	5 (17)	0.74
Fatigue (*n*,%)	7 (23)	8 (27)	0.78
Infusion-related reactions (*n*,%)	6 (20)	6 (20)	1.00
Peripheral neuropathy *(n*,%)	4 (13)	7 (23)	0.33
Nausea (*n*,%)	3 (10)	3 (10)	1.00
Herpes zoster (*n,*%)	4 (13)	2 (7)	0.67
Total events (*n*)	47	51	

Regarding organ toxicity, careful clinical adjudication was performed to distinguish between potential treatment-related effects and progression of underlying amyloidosis. For renal function, One patient in the BD group met criteria for renal progression to end-stage renal disease. In the Dara-based group, one patient developed a transient, reversible episode of acute kidney injury during treatment, which resolved with supportive measures; this was considered possibly related to treatment. Regarding cardiac events, Disease progression leading to fatal heart failure occurred in two patients in the Dara-based group and one patient in the BD group during follow-up. These fatal outcomes were attributed to the natural history of advanced cardiac amyloidosis rather than to direct treatment toxicity.

## 4. Discussion

To reduce treatment burden and minimize toxicity, we developed and evaluated a frequency-adjusted Dara-based regimen. This approach was characterized by: 1) the omission of cyclophosphamide; 2) an adjusted Dara administration frequency (biweekly in the first cycle, then monthly); and 3) a response-guided, individualized treatment duration. Treatment was intended to be discontinued once the patient achieved a hematologic VGPR or better together with a relevant organ response, based on physician assessment and patient agreement, and could be continued for up to 24 months in the absence of disease progression. Our results indicate that this modified approach elicits rapid and deep hematologic and organ responses in patients with newly diagnosed AL amyloidosis, demonstrating its efficacy with a substantially reduced cumulative treatment intensity compared to the standard multiple myeloma-derived Dara schedule [6].

The hematologic efficacy of our frequency-adjusted regimen was both marked and rapid. The significantly higher CR rates at both 6 (57% vs. 27%, *p* = 0.018) and 12 months (63% vs. 27%, *p* = 0.002), coupled with a substantially shortened median time to CR (61 vs. 120 days, *p* = 0.010), demonstrate its superior efficacy compared to BD. The clinical importance of rapid response is underscored by the work of Liu et al. who demonstrated that a “rapid hematologic dFLC response” (reduction ≥67% by cycle 1, day 7) strongly predicts subsequent complete hematologic response and improved organ outcomes [14]. The median time to best hematologic response of 44 days in our cohort suggests that our regimen effectively induces this desirable “rapid responder” phenotype in a majority of patients. Our outcomes reinforce the potent anti-plasma cell activity of Dara-based regimens and are comparable to other real-world studies [15,16].

A key innovation of our cyclophosphamide-free strategy is its ability to achieve excellent outcomes while minimizing treatment burden. The feasibility of omitting alkylators is strongly supported by Shen et al. who reported a ≥ VGPR rate of 67.5% at 3 months with Dara-BD in high-risk patients [7]. Our findings corroborate this approach, demonstrating that a cyclophosphamide-free backbone did not compromise efficacy, as evidenced by significant hematologic and organ response rates (e.g. cardiac: 52%; renal: 56% at 6 months). Beyond reducing clinical treatment burden, optimizing therapeutic efficiency is critical given the considerable economic impact of AL amyloidosis. Real-world analyses indicate that healthcare costs are substantial, largely driven by anti-plasma cell therapies [17]. Our regimen, which achieved deep responses with fewer Dara administrations, aligns with the imperative to develop highly effective yet resource-conscious treatment strategies.

The clinical value of our frequency-adjusted regimen is most compellingly evidenced in the highest-risk setting of Mayo Stage IIIb cardiac amyloidosis. The newly included matched case-control study by Palladini et al. (2025) provides the most robust comparative evidence to date, directly demonstrating that Dara-based regimens significantly prolong overall survival (10.3 vs. 4.0 months) and major organ deterioration-free survival (10.2 vs. 3.2 months) compared to CyBorD in newly diagnosed Stage IIIb patients [5]. This survival benefit was driven by significantly higher rates of deep hematologic response (≥VGPR 41% vs. 16% at 3 months) and cardiac response (25% vs. 3% at 3 months) with Dara. Our study’s findings, showing superior hematologic and organ responses with a Dara-based regimen, are entirely consistent with this landmark analysis and reinforce the imperative to incorporate Dara into frontline therapy for advanced cardiac involvement. The favorable safety profile of our regimen, with no grade 3/4 adverse events and a toxicity spectrum comparable to BD, is a crucial advantage. The omission of cyclophosphamide likely contributed to the low rates of myelosuppression. This is particularly important for Stage IIIb patients, who are exquisitely vulnerable to treatment complications. Oubari et al. also highlighted the superior risk-benefit profile of Dara in this population, observing improved outcomes without an increase in severe toxcitiesi [18].

It should be noted that the shorter median follow-up in the Dara-based group (14 months) compared to the BD group (23 months) reflects its more recent introduction into our clinical practice. Despite robust efficacy, no significant difference in MOD-PFS or OS was observed, which is likely attributable to this follow-up disparity and the profound impact of baseline organ damage on survival. As evidenced by Palladini et al. and Oubari et al. while Dara significantly improves survival in Stage IIIb, early mortality remains high and is often dictated by pre-existing cardiac damage rather than the treatment itself [5,18]. The trend towards a longer duration of complete response (10.8 vs. 8.6 months) in our Dara group is a positive signal that may translate into survival separation with longer observation.

Several limitations of this study should be acknowledged. First, its retrospective, single-center design and modest sample size may limit the generalizability of the findings and the statistical power for survival analyses. Second, the follow-up duration remains relatively short, precluding a definitive assessment of long-term outcomes and response durability after treatment cessation. Third, prognostic molecular cytogenetic data (e.g. +1q21, t(11;14)) were not routinely available, reflecting real-world practice constraints. Despite these limitations, we employed propensity score matching to enhance the robustness of the comparative efficacy analysis. Future prospective studies should integrate early biomarker dynamics, such as the cycle 1, day 7 dFLC response [14], to further refine and validate response-adapted algorithms like our frequency-adjusted regimen.

## 5. Conclusion

Our findings support the use of a frequency-adjusted, cyclophosphamide-free Dara regimen as an effective and safe frontline strategy for AL amyloidosis. This approach maintained high efficacy with substantially reduced treatment intensity, offering a viable and more efficient alternative to conventional, fixed-duration regimens. The compelling efficacy observed in patients with advanced cardiac involvement (Mayo Stage IIIa/IIIb), supported by emerging evidence of survival benefit in this high-risk population, underscores the potential of this strategy to pave the way for a more personalized and less burdensome standard of care.

## Supplementary Material

supplementary figures and tables.docx

## Data Availability

The data that support the findings of this study are available on request from the corresponding author, S.S. The data are not publicly available due to their containing information that could compromise the privacy of research participants.
