# Supplementary material for: Frequency-adjusted daratumumab-based regimen versus bortezomib/dexamethasone in newly diagnosed AL amyloidosis: a matched-cohort study
Source: Ann Med. 2026 Jan 21;58(1):2617767. doi: 10.1080/07853890.2026.2617767 (PMC12825582; doi:10.1080/07853890.2026.2617767)
Supplement: supplementary figures and tables.docx [file IANN_A_2617767_SM9816.docx]

Table S1. Criteria for hematologic response and progression

| Category | Definition |
| --- | --- |
| Complete Response | Negative serum and urine immunofixation and normalization of free light chain levels and ratios* |
| Very Good Partial Response | Baseline dFLC ≥50 mg/L: reduction in dFLC <40 mg/L  Baseline dFLC <50 mg/L: ≥90% reduction in serum M-protein plus urine M-protein <100 mg/24 hours |
| Partial Response | Baseline dFLC ≥50 mg/L: dFLC reduction ≥ 50% from baseline  Baseline dFLC <50 mg/L: ≥50% reduction in serum M-protein plus reduction in 24-hour urine M-protein by ≥90% or to <200  mg/24 hours |
| No Response | Failure to meet criteria for PR, VGPR, or CR |
| Hematologic Progression | Recurrence of an abnormal free light chain ratio with a doubling of the iFLC from complete response  A ≥50% increase in serum iFLC to an absolute value >100 mg/L from any response  50% increase in serum M-protein to >0.5 g/dL or 50% increase in urine M-protein to >200 mg/day from any response |

dFLC denotes difference between involved and uninvolved free light chain. iFLC denotes involved free light chain.

*If the involved FLC is below the upper limit of normal at the time of response assessment, normalization of the uninvolved FLC and the FLC ratio is not required for CR. Baseline measurements are the closest available values prior to the first treatment dose. Definitions are based on International Society of Amyloidosis (ISA) consensus criteria^11, 12^.

Table S2. Criteria for organ response

| Organ | Response Criteria | Progression Criteria |
| --- | --- | --- |
| Kidney | ≥30% decrease in proteinuria or a decrease in proteinuria to <0.5 g/24h in the absence of renal  progression | ≥25% decrease in eGFR |
| Heart | NT-proBNP reduction >30% and an absolute decrease >300 ng/L in patients  with baseline NT-proBNP ≥650 ng/L) or NYHA class response (≥2 class decrease in patients with baseline NYHA class 3 or 4) | NT-proBNP progression (>30% and >300  ng/L increase) or cTn progression (≥33% increase) or ejection fraction progression (≥10% decrease) |

cTn cardiac troponin, eGFR estimated glomerular filtration rate, NT-proBNP N-terminal pro b-type natriuretic peptide, NYHA New York Heart Association.
